# In Silico Preformulation Modeling, Solubility Enhancement, and Sustainable Release of Rebamipide Utilizing Deep Eutectic Mixture Loaded Bioadhesive Controlled Release Granules for Gastritis Treatment

**DOI:** 10.3390/pharmaceutics18050521

**Published:** 2026-04-24

**Authors:** Yasir Qasim Almajidi, Maher Abdulrazzaq Al-Hakeem, Ahmed Yaseen

**Affiliations:** 1 Department of Pharmaceutics, College of Pharmacy, Nahrain University, Baghdad P.O. Box 70064, Iraq; yasir.q.mohammed@nahrainuniv.edu.iq; 2 Department of Pharmaceutics, College of Pharmacy, Gilgamesh University, Baghdad P.O. Box 10021, Iraq; ahmed.y.alsharea@gu.edu.iq

**Keywords:** Rebamipide, deep eutectic mixture (DEM), solubility enhancement, bioadhesive granules, controlled release, mucoadhesion, gastroretention

## Abstract

**Background/Objectives:** Rebamipide is a gastroprotective agent with poor aqueous solubility and rapid gastrointestinal clearance, leading to reduced therapeutic efficiency. This study aimed to enhance the solubility, mucoadhesion, and sustained oral delivery of Rebamipide through the development of a deep eutectic mixture (DEM)-based bioadhesive controlled-release granule formulation. **Methods:** In silico hydrogen-bonding interactions between Rebamipide, malonic acid, and urea were analyzed using CCDC tools. A thermodynamically stable DEM (1:3:1) was prepared and incorporated into bioadhesive granules using chitosan and HPMC. Physicochemical characterization was conducted using FTIR, DSC, TGA, and PXRD. Solubility, in vitro dissolution, ex vivo mucoadhesion (sheep gastric mucosa), and in vivo gastric retention (BaSO_4_-loaded granules in rats) were evaluated. **Results:** The optimized DEM significantly enhanced Rebamipide solubility (10.08 mg/mL vs. 0.045 mg/mL). Solid-state analyses confirmed hydrogen-bond formation and reduced crystallinity. DEM granules exhibited sustained drug release over 24 h (99.7 ± 0.8%) with improved dissolution efficiency compared to the marketed tablet (Mucosta®, 100 mg; T_50_%: 5.03 h vs. 0.82 h). Kinetic modeling indicated non-Fickian anomalous transport (*n* = 0.47). The bioadhesive force of DEM granules (0.29 ± 0.02 N) was significantly higher than that of the pure drug and physical mixture. In vivo radiographic studies confirmed prolonged gastric retention. **Conclusions:** The DEM-based bioadhesive granule system effectively improves solubility, dissolution rate, mucoadhesion, and gastric retention of Rebamipide. This approach represents a promising platform for once-daily gastroretentive oral delivery, pending further pharmacokinetic evaluation.

## 1. Introduction

Gastritis, one of the most prevalent gastric inflammatory diseases, can lead to serious complications if there is no timely diagnosis and treatment. Proper therapy requires an effective targeted therapy approach [1]. Unfortunately, many drugs offer limited effective therapy because of their solubility and rapid gastric clearance [2]. Rebamipide, a gastroprotective and anti-inflammatory cytoprotective agent, demonstrates low bioavailability because of limited aqueous solubility, thus hindering localized gastric therapy [3].

In recent years, advanced drug delivery systems have sought to optimize drug formulations to achieve greater therapeutic outcomes in drug solubility and gastric retention. Of these, deep eutectic mixtures (DEMs) have increasingly gained attention for their ability to enhance solubility in poorly soluble therapeutics [4]. The present study utilized a novel DEM of urea and malonic acid to achieve greater solubility of Rebamipide. The solubilization mechanism was explained using hydrogen bond interaction data from the CCDC, which demonstrated the existence of strong and thermodynamically favorable hydrogen bonds between the drug and the eutectic components [5]. The selection of urea and malonic acid as DEM components was based on several formulation and mechanistic considerations. Both materials are pharmaceutically acceptable and possess well-established hydrogen-bond donor–acceptor properties that favor eutectic formation with poorly soluble drugs. Urea is a bifunctional molecule containing NH_2_ donor groups and a carbonyl acceptor group, enabling versatile intermolecular interactions with Rebamipide functional groups such as phenolic OH, amide NH, and carbonyl oxygen atoms. Malonic acid was selected because its dicarboxylic structure provides a higher density of hydrogen-bonding sites than monocarboxylic acids such as acetic acid or lactic acid, thereby increasing the likelihood of forming a cooperative hydrogen-bonding network capable of destabilizing the crystalline lattice of Rebamipide. Compared with other commonly reported eutectic-forming materials such as choline chloride, citric acid, or nicotinamide, the urea–malonic acid combination was considered more suitable for the present system because it offers a balanced combination of strong intermolecular interaction potential, good miscibility, and mild processing requirements. In addition, both components can form eutectic systems at relatively low temperatures (60–70 °C), which minimizes the risk of thermal degradation of Rebamipide during preparation. Thus, the selection of these two components was guided not only by availability and safety, but also by their expected ability to maximize hydrogen-bond-mediated solubilization and amorphization of the drug.

Other than enhancing solubility, mucoadhesive controlled-release granules containing HPMC (hydroxypropyl methylcellulose) and chitosan were developed to sustain drug release and extend gastric residence time [6]. These polymers possess desirable adhesive and swelling properties, which are essential in maintaining the localization of the drug in the gastric environment [7]. Mercury and Conquest™ software suite provided guidance for in silico preformulation modeling, which helped in rational mechanistic formulation development [8]. This modeling provided information on pre-simulated gastrointestinal, solubility, and absorption, dissolution, and absorption geometry, and dissolution and absorption kinetics. The combination of rational mechanistic development and empirical data from CCDC formulates the development from a rational mechanistic perspective [9]. The developed strategy in this study is the use of hydrogen bond solubilization and gastric retention. This introduces a scalable and mechanistically grounded approach for potentially improving the oral bioavailability of Rebamipide, pending formal pharmacokinetic confirmation. The outcomes not only contribute to the improved management of gastritis but also provide a platform for addressing solubility challenges in other BCS Class II/IV drugs using DEM-based and mucoadhesive technologies.

## 2. Materials and Methods

### 2.1. Materials

Rebamipide (purity ≥ 98%) was kindly supplied by Koye Pharmaceuticals Pvt. Ltd. (Mumbai, India). Sigma-Aldrich (St. Louis, MO, USA) provided the malonic acid (purity ≥ 98%) and urea (analytical grade, ≥99%). The supplier of hydroxypropyl methylcellulose (HPMC, K100M grade) was Colorcon Asia Pvt. Ltd. (Goa, India), and chitosan was obtained from Lubrizol Advanced Materials Inc. (Cleveland, OH, USA). Ethanol (absolute), methanol (HPLC grade), potassium dihydrogen phosphate (KH_2_PO_4_), sodium hydroxide pellets, and additional analytical-grade reagents and solvents used in this research were gifted by Pioneer Company for Scientific Supplies, Sulaymaniyah, Iraq. The commercially available Rebamipide product used as a comparator in dissolution studies was Mucosta^®^ tablets (100 mg Rebamipide per tablet; Otsuka Pharmaceutical Co., Ltd., Tokyo, Japan), purchased from a licensed local pharmacy. Each tablet was used as supplied, without further modification, and an amount equivalent to 100 mg Rebamipide was employed per dissolution run to ensure equivalent dose comparison with the prepared DEM granules. Chemical structures were drawn using ChemDraw software (version 20.0, PerkinElmer Informatics, Waltham, MA, USA). DDsolver (Microsoft Excel add-in, version 1.0) was used for in vitro release modeling and analysis. ConQuest (v2.0.5, CCDC, Cambridge, UK) and Mercury (v4.3.0, CCDC, Cambridge, UK) software were used for Hydrogen bond computation prediction. GraphPad Prism (version 9.0, GraphPad Software, San Diego, CA, USA) was used for statistical analysis.

### 2.2. Methods

#### 2.2.1. Validation of UV–Visible Spectrophotometric Method

The UV–Visible spectrophotometric method used for quantification of Rebamipide at λmax = 226 nm was validated in accordance with ICH Q2(R1) analytical validation guidelines. Linearity was established over a concentration range of 1–20 µg/mL, demonstrating a strong linear relationship between absorbance and concentration (R^2^ = 0.9994; regression equation: y = 0.0423x + 0.0061). Beer–Lambert law was obeyed throughout the working range. The limit of detection (LOD) and limit of quantification (LOQ) were calculated using the formulae LOD = 3.3σ/S and LOQ = 10σ/S, where σ is the standard deviation of the intercept and S is the slope of the calibration curve, yielding values of 0.28 µg/mL and 0.84 µg/mL, respectively. Precision was assessed through intra-day (3, same day) and inter-day (*n* = 3, three consecutive days) studies at three concentration levels (5, 10, and 15 µg/mL). Intra-day RSD values ranged from 0.41% to 0.87%, and inter-day RSD values ranged from 0.63% to 1.14%, both well within the ICH-accepted threshold of ≤2%, confirming good repeatability and intermediate precision. Accuracy was evaluated via recovery studies at 80%, 100%, and 120% of the nominal concentration, yielding mean percentage recoveries of 99.4 ± 0.6%, 100.1 ± 0.5%, and 99.8 ± 0.7%, respectively, all within the accepted range of 98–102%. Specificity was confirmed by the absence of any significant spectral interference from excipients (HPMC K100M, chitosan, MCC, magnesium stearate), DEM components (malonic acid, urea), or dissolution media (0.1 N HCl, pH 1.2) at 226 nm. The validated method demonstrated adequate sensitivity, specificity, linearity, precision, and accuracy for reliable quantification of Rebamipide across all solubility, drug content uniformity, and dissolution studies in this work.

#### 2.2.2. Computational Prediction of Hydrogen Bonding Using CSD Tools

**Figure 1:**
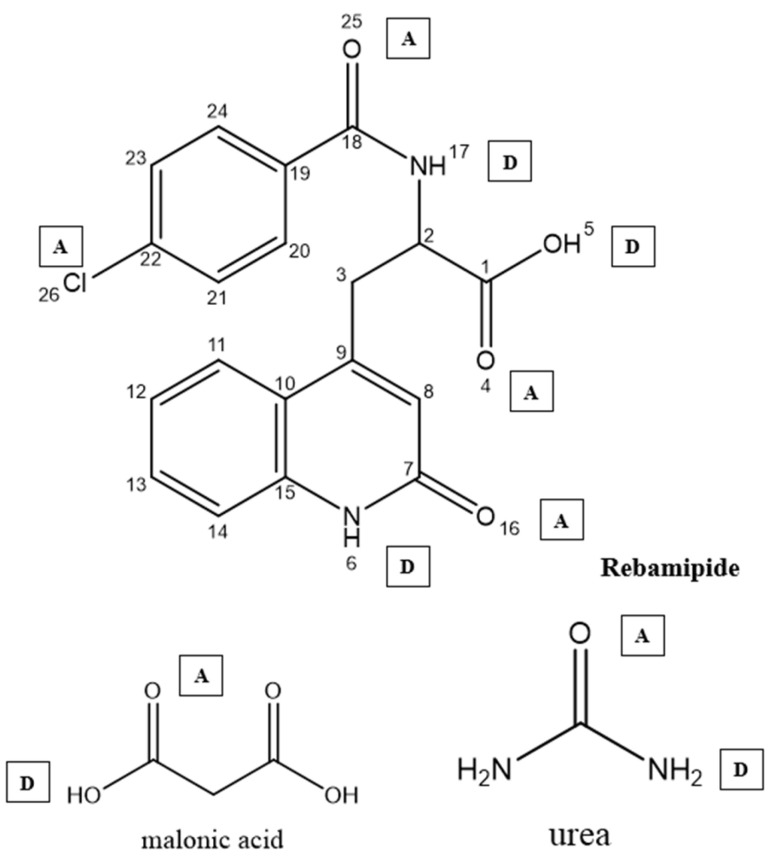
The chemical structures of Rebamipide, malonic acid, and urea using ChemDraw software (version 20.0, PerkinElmer Informatics, Waltham, MA, USA), (note: atomic numbering according to eye view, D and A defined as hydrogen bond donor–acceptor respectively).

Two software tools from the Cambridge Crystallographic Data Centre [ConQuest (v2.0.5, CCDC, Cambridge, UK) and Mercury (v4.3.0, CCDC, Cambridge, UK)] were used to foresee possible H-bonding associations within molecules. ConQuest is the Cambridge Crystallographic Database search engine, and users can construct complex queries to extract specific crystallographic information. In addition, users can search for certain bond and geometric arrangements [10]. Mercury, on the other hand, permits the visualization and manipulation of 3D representations of crystal forms and their structures. This helps one to understand their characteristics better. For the analysis of interrelated molecular structures, there are integrated software tools for data processing, which include spreadsheets, statistical graphs, structural overlays, and other means [11]. The computational prediction of hydrogen bonding was performed as follows (Table 1): the functional groups of Rebamipide (phenolic OH, amide NH, carbonyl oxygens O4, O16, O25), urea (NH_2_ donor, C=O acceptor), and malonic acid (carboxylic OH donors, carbonyl C=O acceptors) were defined as hydrogen bond donor–acceptor queries in ConQuest (Figure 1). The CSD was searched for crystal structures containing geometric arrangements matching these functional group pairs, with a maximum D–H⋯A distance of 3.5 Å and a minimum D–H⋯A angle of 90° as search filters, consistent with established hydrogen bond geometry criteria [12,13]. The number of CSD structural hits, the mean H-bond length (Å), and the mean H-bond angle (°) were recorded for each donor–acceptor pair as indicators of interaction probability and geometry quality. Mercury software was used to visualize the predicted intermolecular arrangement and generate the molecular interaction diagram (Figure 1). A high hit count combined with short H-bond length (<2.1 Å) and near-linear angle (>150°) was used as the criterion for classifying an interaction as strong and thermodynamically favorable [12].

Strong, directional hydrogen bonding interactions were predicted computationally between the –NH and –OH groups of Rebamipide and the carbonyl and hydroxyl groups of urea and malonic acid. This indicates the likely formation of a stable ternary hydrogen-bonded system, which should enhance the drug’s physicochemical properties. The analysis results, in the form of predicted hydrogen bond lengths and angles, were described in Table 1.

#### 2.2.3. Preparation of Deep Eutectic Mixtures (DEMs)

To improve the solubility of Rebamipide, deep eutectic mixtures (DEMs) were formulated using urea and a donor of hydrogen bonds and receptor components. Three different ternary formulations were analyzed to choose the best eutectic composition for the solubility enhancement. The following molar ratios were selected.

1:2:1—Rebamipide:Malonic Acid:Urea
1:1:2—Higher urea content
1:3:1—Higher malonic acid content

The reason behind the selection of these ratios was to explore the effects of the ratio of hydrogen bond donors/acceptors on the formation of eutectics and thermodynamic stability, and subsequent enhancement of the solubility of Rebamipide [14]. Urea and malonic acid were prioritized over other potential eutectic-forming candidates because their complementary donor–acceptor functionalities and bifunctional architectures were expected to provide stronger and more cooperative intermolecular interactions with Rebamipide than simpler monofunctional or less geometrically compatible alternatives. The DEMS was prepared following a four-step standard protocol.

Dry Mixing: To obtain homogeneous distribution, the weighed parameters were mixed precisely using a mortar and pestle.

Gentle Heating: The dry mixtures were then added to the sealed vials and placed in the water bath at 60–70 degrees Celsius, which induced eutectic fusion with continuous stirring.

Cooling and Stabilization: The molten products were left to cool slowly to room temperature, and this stabilized the molten systems. Depending on the ratio and interactions, the systems had stable eutectic liquids or soft amorphous solids. DEMs were prepared and kept at room temperature and refrigerated conditions for 48 h to evaluate physical and phase stability. The systems that retained their physical conditions in both conditions were considered to further develop the formulations.

#### 2.2.4. Characterization of DEMs

##### Saturated Solubility Determination of Rebamipide in Prepared DEMs

Rebamipide saturated solubility was determined in three deep eutectic mixtures (DEMs) that were made using various molar ratios of Rebamipide:Malonic Acid:Urea (1:2:1), (1:1:2), and (1:3:1). A total of 5 mL of each formulation was placed in individual glass vials, and an excess of Rebamipide was added to each vial. The vials were placed on a magnetic stirrer to mimic room temperature and held at 25 °C for 48 h to allow sufficient time for saturation equilibrium [15]. After stirring, samples were filtered through 0.45 μm syringe filters and diluted with methanol for later determination of Rebamipide concentration. Absorbance was measured at λmax = 226 nm using a previously validated UV–Visible spectrophotometric method, ensuring accuracy, precision, and sensitivity within the working concentration range, and all readings were averaged from three separate measurements. These solubility values were compared to the pure aqueous solubility to assess the solubility enhancement for each of the three DEM formulations. The implementation of the DEM that provided the greatest aqueous solubility was selected for further characterization.

##### Structural Characterization by FTIR Spectroscopy

Using Fourier-transform infrared (FTIR) spectroscopy, potential molecular interactions, and hydrogen bonds between Rebamipide and the different molar ratio, deep eutectic mixtures (DEMs) were analyzed. Rebamipide was analyzed alone, as were the different Rebamipide-loaded DEM formulations [(1:2:1), (1:1:2), and (1:3:1); Rebamipide:Malonic Acid:Urea]. These samples were individually analyzed and then compacted into uniform discs with dry potassium bromide (KBr) using a manual hydraulic press. For this analysis, a Shimadzu FTIR-8400S Spectrophotometer was used, and the IR spectra were generated from 4000–400 cm^−1^ [16]. Spectral analysis was directed towards identifying the shifts and distortions of the characteristic absorption bands of the potential hydrogen bonds of the interacting functional groups, like -NH, -OH, C=O, and COOH, as a way of identifying the intermolecular interaction of Rebamipide with the components of the DEM.

##### Selection of the Optimal DEM Formulation

The selection of the best deep eutectic mixture (DEM) formulation for Rebamipide was primarily based on maximum aqueous solubility and the presence of molecular interactions confirmed via FTIR spectroscopy. From the prepared molar ratios, the formulation with the highest drug solubility and prominent spectral shifts suggestive of hydrogen bonding was recognized as the most appropriate. This selected DEM formulation (considering the highest aqueous solubility) was then used for the subsequent development of bioadhesive controlled-release granules for oral administration.

##### Thermal Behavior and Stability Analysis of Rebamipide and Selected DEM Using DSC and TGA

To examine Rebamipide and DEM formulations’ thermal behavior and stability, from the ideal formulations based on aqueous solubility, Thermogravimetric Analysis (TGA) and Differential Scanning Calorimeter (DSC) were used. These are used to assess the various stages of pharmaceutical formation, possible molecular interactions, and the decomposition of the formulation [17]. For the selected DEM formulation, approximately 5–10 mg of Rebamipide was carefully measured with the same material placed in hermetic aluminum pans, which were sealed at the edges. For both the DSC and TGA runs, the same material was used with a Mettler Toledo DSC823e device (Mettler-Toledo, Greifensee, Switzerland). To prevent oxidative degradation, instruments were set to sample temperatures of 40 degrees Celsius to 350 degrees Celsius with a 10 degrees Celsius/min increase. Nitrogen was used to treat and eliminate the samples. The DSC thermograms showed a melting point depression, glass transition, and the disappearance or shifting of thermal events, which indicated the formation of an eutectic system and possible hydrogen bonding of Rebamipide with the eutectic components. TGA curves were employed to identify the onset of thermal degradation, mass loss phases, and resulting content, which proved the thermal stability of the chosen eutectic system. The combination of the DSC-TGA readings provided vital information on the physicochemical compatibility and thermal applicability of Rebamipide in the eutectic matrix that could be further processed into a pharmaceutical product [18,19].

##### Powder X-Ray Diffraction (PXRD) Analysis of Rebamipide and Selected DEM

To identify the crystalline form of Rebamipide in the chosen DEM structure (according to the maximum aqueous solubility), PXRD analysis was undertaken. Diffraction patterns of pure Rebamipide and the Rebamipide-loaded DEM (based on the highest aqueous solubility) were recorded using a Bruker D8 Advance diffractometer (Billerica, MA, USA) running at 40 kV and 40 mA, using Cu-Kα radiation (λ = 1.5406 Å). At a step size of 0.02°/s, scans were conducted throughout a 2θ range of 5° to 80° [20]. The crystalline peaks of pure Rebamipide were compared with those of the selected DEM formulation. Reduction in characteristic diffraction peaks considered as evidence of amorphization or molecular dispersion of the drug within the eutectic matrix, supporting its potential for enhanced solubility and pharmaceutical performance [21].

##### Preparation of Rebamipide-Loaded DEM as Bioadhesive Controlled-Release Granules

The wet granulation technique [22] was employed to prepare bioadhesive controlled-release granules containing the selected Rebamipide-loaded deep eutectic mixture (DEM), intended for oral administration via capsule filling. A quantity of the selected DEM equivalent to 100 mg of Rebamipide was geometrically blended with chitosan (as a bioadhesive polymer), HPMC K100M (for sustained release), and microcrystalline cellulose (MCC) as a diluent and flow enhancer. Aerosil helps with the flow of the powder, whereas magnesium stearate and talc serve as a lubricant and glidant, respectively. The complete composition is in Table 2.

To obtain the cohesive wet mass and granules, a small quantity of ethanol–water mixture was added to the dry blend. The material was then passed through a 2 mm sieve in order to obtain homogeneous granules. The granules were then dried in a hot air oven for 30 min at 40 °C. The dried granules were then further resized using mesh #20 in order to achieve uniform particle distribution. Approximately 500 of the dried granules, which are 100 mg of Rebamipide, were packed into firm gelatin capsules size 0. The capsules were kept in airtight containers with desiccant at room temperature until further evaluation.

To isolate the specific contribution of the deep eutectic mixture (DEM) to the observed dissolution performance, a DEM-free matrix control formulation (F-Control) was prepared in parallel. F-Control granules were manufactured using an identical wet granulation process and excipient composition as the optimized DEM granules, with the sole modification of substituting the DEM with an equivalent mass of pure crystalline Rebamipide (100 mg drug loading), while maintaining identical proportions of HPMC K100M, chitosan, MCC, aerosil, magnesium stearate, and talc (Table 2). This design ensures that any difference in dissolution behavior between F-Control and DEM granules is exclusively attributable to the DEM and not to matrix composition or processing variables. F-Control granules were subjected to the identical dissolution test conditions described above (USP Type II paddle apparatus, 900 mL of 0.1 N HCl pH 1.2, 37 ± 0.5 °C, 50 rpm, *n* = 3), and cumulative percentage drug release was calculated at the same time intervals.

##### Physical Characterization of Prepared Granules

The bioadhesive regulated-release pellets containing the selected Rebamipide-loaded deep eutectic mixture (DEM) were evaluated for essential pre-compression attributes [23]. The flowability was evaluated by means of the angle of repose and the calculation of bulk and tapped densities, Carr’s index, and Hausner’s ratio. Sieve analysis was performed to ascertain the particle size for the granule encapsulation uniformity. The moisture content was determined through the loss on drying method, and the friability method was employed to determine the mechanical strength for storage and transport. UV spectrophotometry was performed and assessed at 226 nm with the mean and standard deviation for uniformity of the drug content. The granules displayed adequate flow, stability, and content uniformity for filling the capsules. All the evaluations were done, as these findings demonstrate.

##### Swelling Index Determination

The swelling capacity of hydration was used to identify the granules with a potential for sustained release and mucoadhesion. A total of 500 mg of granules, which are the contents of one capsule, were placed in pre-weighed tea bags and submerged in 100 mL of 0.1 N HCl (pH 1.2) and kept at 37 ± 0.5 °C [24]. At predetermined intervals (1, 2, 4, 6, 12, and 24 h), the tea bags were removed, gently blotted with filter paper to remove surface moisture, and reweighed. The swelling index (SI) was calculated using the formula:

$$Swelling\;Index\left(\%\right)=\frac{W_{t}-W_{0}}{W_{0}}\times 100$$

where

- *W*_0_ = initial weight of the dry granules
- *W_t_* = weight of swollen granules at time *t*

The test was run in triplicate, and the mean ± SD was used to represent the findings.

##### Matrix Erosion Study

To assess the matrix erosion behavior of the granules in acidic medium, pre-weighed samples of granules (500 mg) were enclosed in pre-dried and pre-weighed tea bags and placed in 100 mL of 0.1 N HCl (pH 1.2) at 37 ± 0.5 °C under gentle agitation (50 rpm) [25].

At specific time intervals (1, 2, 4, 6, 12, and 24 h), the tea bags were removed, washed with distilled water to eliminate dissolved drug residues, dried at 40 °C for 24 h, and reweighed.

The percentage matrix erosion was calculated as

$$Matrix\;Erosion\;\left(\%\right)=\frac{W_{0}-W_{d}}{W_{0}}\times 100$$

where:

- *W*_0_ = initial weight of the dry granules
- *W_d_* = weight of dried granules after exposure

Each measurement was performed in triplicate, and results were reported as mean ± SD.

##### In Vitro Dissolution Study

To assess the comparative performance the in vitro release profile of Rebamipide from the prepared DEM granule-loaded capsule, the control formulation (F-Control), and the marketed tablet Mucosta^®^ tablets (Rebamipide 100 mg; Otsuka Pharmaceutical Co., Ltd., Tokyo, Japan) was evaluated. Using a USP type II (paddle) equipment (Electrolab, Mumbai, India, TDT-06T), dissolution testing was carried out using 900 mL of 0.1 N HCl (pH 1.2) as the dissolution medium, which was kept at 37 ± 0.5 °C and agitated at 50 rpm. Five milliliter samples were taken out at prearranged intervals and promptly replaced with an equivalent amount of new medium. After passing through a 0.45 µm membrane filter and being diluted with 0.1 N HCl, the collected samples were examined at λmax 226 nm using a validated UV–Visible spectrophotometric method, with established linearity, precision, accuracy, and limit of quantification suitable for dissolution analysis [26]. Mean ± standard deviation (*n* = 3) was used to indicate the cumulative percentage of drug release from both the produced granules and the marketed tablet. The kinetic fit model and comparative dissolution profiles were analyzed by DDsolver software (Microsoft Excel add-in, version 1.0) using key parameters such as dissolution efficiency (DE%), T_50_%, and similarity factor (f_2_) to evaluate the extent of release enhancement provided by the optimized DEM-based formulation [27].

##### Ex Vivo Bioadhesive Strength Determination

**Figure 2:**
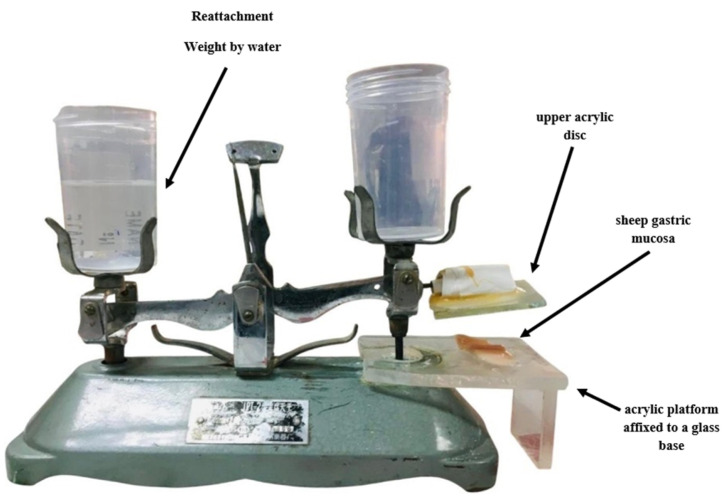
The modified physical balance apparatus was utilized for Ex Vivo Bioadhesive Strength Deter-mination.

A modified physical balance device was used to assess the bioadhesive strength of the prepared Rebamipide DEM granules (Figure 2). In this configuration, the right pan of a conventional two-pan mechanical balance was replaced with an acrylic platform attached to a glass base, onto which sheep gastric mucosa was attached. Sheep gastric mucosa was chosen as a model for mucoadhesion studies because of its anatomical and physiological resemblance to human gastric tissue. Adhesive tape was used to attach a representative number of granules (compressed or cohesive) to an upper acrylic disc that was attached to the beam’s arm. The mucosal surface was moistened with 100 µL of 1% *w*/*v* mucin solution prior to the experiment. The granule-bearing disc was gently brought into contact with the mucosa and maintained under slight pressure for 180 s. Then, water was gradually added to the left pan until the detachment of the granule occurred [28]. The mass required to break the adhesive bond was recorded and converted to detachment force (N) using the formula:


$$Force\;\left(N\right)=\frac{Mass\;(g)\times 9.81}{1000}$$

##### In Vivo Radiographic Studies

The radiological study protocol was approved by the Scientific Committee of Al-Mustansiriyah University, Baghdad, Iraq (Approval Code: BCSMU/0122/0004Z) and overseen by a qualified expert in animal welfare. In this study, Male Sprague–Dawley rats (RRID: RGD_70508) were randomized into four groups (*n* = 6 per group), each weighing approximately 300 g and aged four months, and were utilized. Before the experimental procedures, the rats underwent a one-week habituation phase under controlled environmental conditions, including a regulated 12 h light/dark cycle, a stable temperature of 25 °C, and unrestricted access to both water and a balanced diet.

In this study, the X-ray technique was used to determine the gastric residence time of the prepared granules. BaSO4 was used to make the granules X-ray opaque. For this study, 75 mg of BaSO4 was loaded in the prepared bioadhesive controlled-release granules containing the selected DEM (15 mg granules equivalent to 3 mg drug were suspended in 2.5 mL of 15% BaSO_4_ suspension. The study was conducted under the guidance of an expert radiologist. After overnight fasting, the rats were fed with a powdered soy diet. Half an hour later, a BaSO_4_-loaded prepared granule was given to every rat via oral plastic gavage tube (15 G, 100 mm). The procedure was carried out following approximately 3 min of isoflurane anesthesia induced in a chamber, whereby gavage was performed upon recovery of the swallow reflex. At different time intervals like 1, 6, 12, and 24 h, the live rats were exposed to abdominal X-ray imaging in a standing position using a portable X-ray unit (Porta 100HF 2.0 kW high frequency; Job Corporation, Yokohama, Japan). The distance between the source of X-rays and the subject was kept constant for all images. Thus, the observation of the prepared granules’ movements could be easily noticed [29]. The mean gastric residence time was calculated.

## 3. Results and Discussion

### 3.1. Computational Prediction of Hydrogen Bonding Using CSD Tools

To understand the potential of hydrogen bond formation between Rebamipide and selected hydrogen bond donors/acceptors used in the deep eutectic mixture (DEM), an in silico screening was conducted using the Cambridge Structural Database (CSD) via ConQuest and Mercury software. The predicted lengths, angles, and sites of intermolecular hydrogen bonds confirmed the rational design of eutectic solvents to improve solubility. To provide a more quantitative interpretation of the intermolecular interactions, hydrogen bonding parameters obtained from the CSD analysis were further evaluated in terms of interaction geometry and frequency. Short hydrogen bond distances (<2.1 Å) combined with near-linear bond angles (>150°) are widely recognized indicators of strong and energetically favorable interactions. In addition, the high frequency of occurrence (CSD hit counts exceeding several hundred instances) suggests that these interaction motifs are thermodynamically preferred in crystalline environments. Collectively, these semi-quantitative descriptors support the formation of a stable hydrogen-bonded network within the ternary system, contributing to lattice disruption, reduced crystallinity, and enhanced solubilization of Rebamipide.

### 3.2. Hydrogen Bonding with Malonic Acid

**Figure 3:**
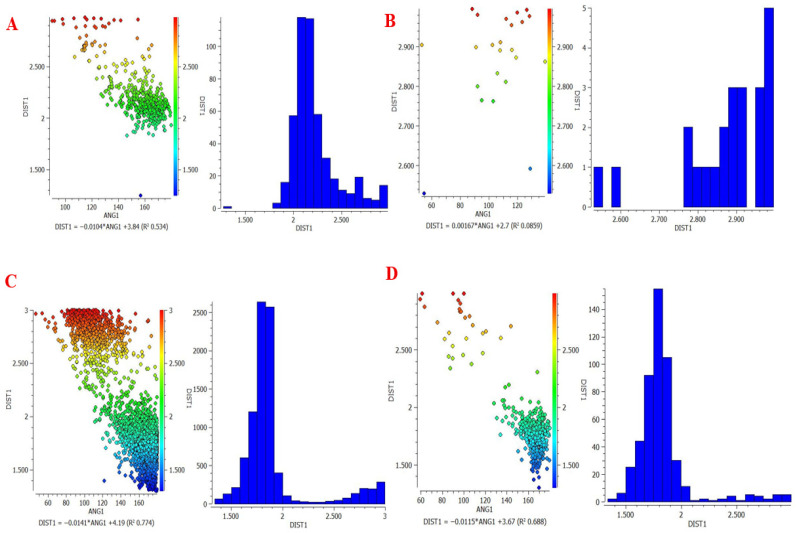
Scatterplot of distance (DIST 1) versus angle (ANG) and histogram for H-bond between (**A**) NH17-O=C of Malonic acid, (**B**) C–Cl (aryl chloride substituent)-HO of Malonic acid, (**C**) OH5-O=C of Malonic, and (**D**) O25-HO of Malonic acid, DIST 1 = H⋯A hydrogen bond distance (Å); ANG = D–H⋯A bond angle (°).

Rebamipide formed many strong hydrogen bonds with the -OH and C=O functional groups with malonic acid (Table 3, Figure 3).

The combination of high interaction frequency, short bond lengths (≈1.8–2.2 Å), and near-linear bond angles (>150°) provides strong semi-quantitative evidence of energetically favorable hydrogen bonding, which is expected to significantly contribute to lattice destabilization and enhanced solubility [30].

### 3.3. Hydrogen Bonding with Urea

**Figure 4:**
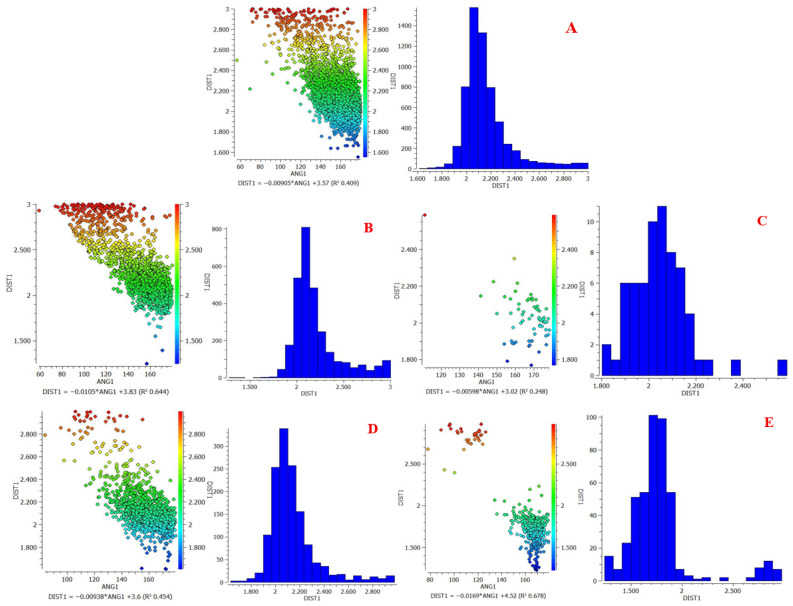
Scatterplot of distance (DIST 1) versus angle (ANG) and histogram for H-bond between (**A**) O25-HN of urea, (**B**) O4-HN of urea, (**C**) O16-HN of urea, (**D**) NH17-O=C of urea, and (**E**) OH5-O=C of urea, DIST 1 = H⋯A hydrogen bond distance (Å); ANG = D–H⋯A bond angle (°).

Urea displays stable hydrogen bonds with Rebamipide, attributable to its annotative bifunctional NH_2_ and C=O groups (Table 4, Figure 4).

The quantitative CSD-derived parameters (high hit counts, optimal bond lengths, and favorable angular geometry) further confirm the cooperative role of urea in stabilizing the hydrogen-bond network, thereby enhancing the overall solubilization efficiency of the ternary eutectic system [31].

**Figure 5:**
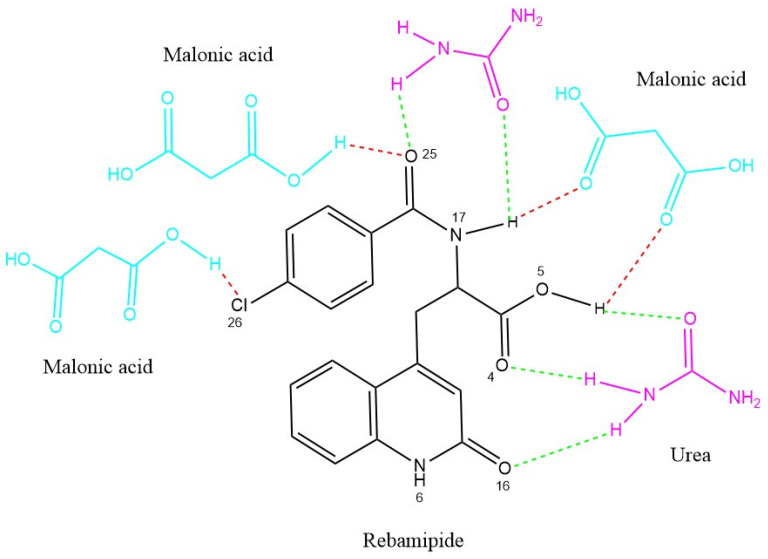
Estimated molecular interaction diagram of the eutectic mixture showing possible hydrogen bonding between Rebamipide, Malonic acid, and Urea. The molecular structures of Rebamipide (black), Malonic acid (cyan), and Urea (magenta) are displayed with colored dashed lines indicating predicted hydrogen bonds based on CCDC analysis.

The DEM 1:3:1 was further supported with CSD-based computational hydrogen bond analysis referenced with CSD via ConQuest and Mercury for the computational work. The predicted interactions reveal multiple short, near-linear hydrogen bonds between Rebamipide’s phenolic OH (O5), amide NH (N17), and carbonyl oxygen (O25) with the carboxylic OH groups of malonic acid and the C=O/NH_2_ groups of urea. The estimated arrangement of these interactions is illustrated in Figure 5, where red dashed lines indicate Rebamipide–Malonic acid hydrogen bonds, and green dashed lines represent Rebamipide–Urea interactions. This cooperative hydrogen-bond network likely facilitates extensive lattice disruption, improved wettability, and formation of a microenvironment that stabilizes Rebamipide in solution [32].

### 3.4. Saturated Solubility Determination of Rebamipide in Prepared DEMs

**Figure 6:**
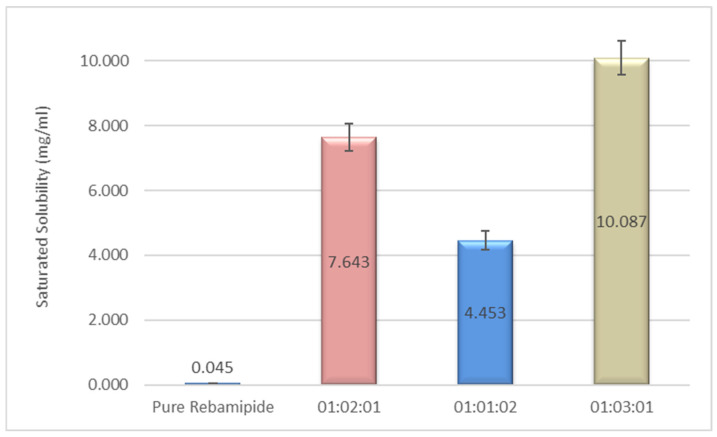
Saturated solubility of pure Rebamipide and DEM formulations (mean ± SD, *n* = 3).

The saturated solubility of pure Rebamipide and three prepared deep eutectic mixtures (DEMs) with varying molar ratios of Rebamipide:Malonic acid:Urea was evaluated at 37 ± 0.5 °C. As shown in Figure 6, pure Rebamipide exhibited an extremely low aqueous solubility of 0.045 mg/mL, consistent with its classification as a poorly water-soluble drug (BCS Class IV). Incorporation into Drug-Excipient Microparticles (DEMs) led to an increase in drug solubility. With a drug-to-urea-to-malonic acid ratio of 1:2:1, we achieved a solubility of 7.643 mg/mL, which is an increase of almost 170 times the solubility compared to the pure drug. 1:1:2 urea to drug to malonic acid had less of an increase at 4.453 mg/mL, which indicates that a higher ratio of urea alone was not as effective. The other noted composition of 1:3:1 was able to achieve a solubility of 10.087 mg/mL, which is a more than 220-fold increase compared to the pure Rebamipide.

The substantial enhancement in Rebamipide solubility observed upon formation of deep eutectic mixtures (DEMs), particularly at the 1:3:1 molar ratio, can be attributed to a synergistic hydrogen-bonding network and pronounced disruption of the drug’s crystalline lattice. The superior performance of the 1:3:1 formulation, compared to other molar ratios, indicates a critical compositional balance in which malonic acid functions as a dominant structural modulator within the ternary system. Its bifunctional carboxylic groups enable the formation of a dense and cooperative hydrogen-bonding network, as supported by semi-quantitative CSD-derived parameters, including high interaction frequencies, short hydrogen bond distances (<2.1 Å), and near-linear bond angles (>150°), which collectively indicate energetically favorable intermolecular interactions. These interactions promote lattice destabilization and amorphization, as further corroborated by FTIR spectral shifts and molecular modeling analyses [33]. The strong bonding to the 1:3:1 ratio also guides the subsequent characterization of 1:3:1 DEM in DSC, TGA, PXRD, and bioadhesive controlled-release granule formulations.

### 3.5. Structural Characterization by FTIR Spectroscopy

**Figure 7:**
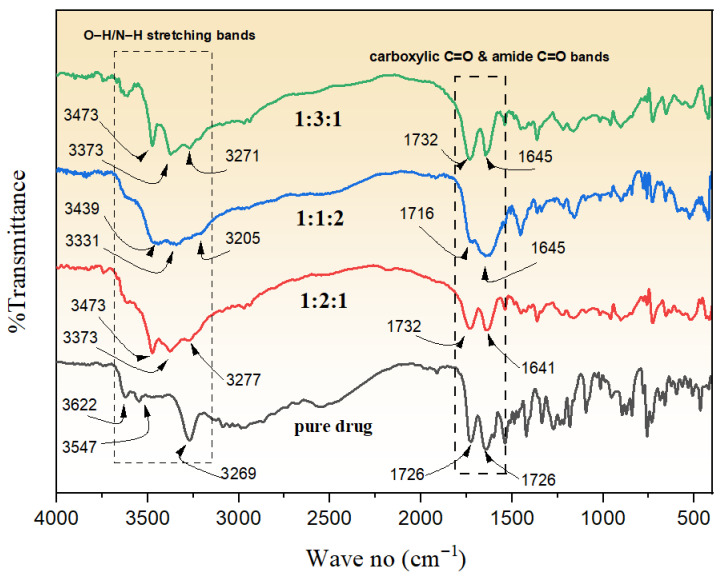
FTIR spectrum of pure Rebamipide compared to the prepared DEMs.

The FTIR analysis portrays unique patterns for Rebamipide in addition to its deep eutectic mixtures in 1:2:1, 1:1:2, and 1:3:1 combinations with malonic acid and urea, suggesting significant intermolecular interactions, as presented in Figure 7. Pure Rebamipide displayed characteristic O–H/N–H stretching bands at 3622, 3547, and 3269 cm^−1^, a sharp carboxylic C=O peak at 1726 cm^−1^, an amide C=O at 1643 cm^−1^, and well-defined aromatic/amide II bands at 1540, 1487, and 1467 cm^−1^ [34]. There were notable changes in the 1:2:1 DEM. The appearance of the O–H/N–H region at 3473, 3373, and 3277 cm^−1^ at diminished intensity reflected the formation of more robust hydrogen bonds. As for the 1:1:2 DEM, peaks were recorded at 3439, 3331, and 3205 cm^−1^. In comparison to the 1:2:1 system, the peaks were less broadened, indicating a weaker hydrogen bond network, most probably as a result of Urea’s excess and its interference with optimal ternary association. C=O peaks at 1716 and 1645 cm^−1^ were less prominent in overlap compared to 1:2:1, which suggests that the 1:1:2 DEM had weaker interactions. For 1:3:1 DEM, 3473, 3373, 3271 cm^−1^ were recorded, and the Overall C=O region at 1732, 1645 cm^−1^ displayed the greatest broadening, while in the H-bond region of the spectrum, intensity was reduced to the greatest extent. The carbonyl bands kept shifting more downfield, partly merging, reflecting more H-bonding as a result of the increased malonic acid content. This malonic acid, with its more acidic protons, readily interacted. Fingerprint region shifts (e.g., 1363, 1219, 958, 902, 729 cm^−1^) further supported structural modification at the molecular level [35,36]. The observed solubility patterns for the 1:3:1 DEM, with 1:2:1 following, and 1:1:2 DEM in decreasing order of solubility are thus explained by these spectral changes. The 1:3:1 system’s FTIR features with pronounced shifts attributable to stronger hydrogen bonding and C=O peak merging validate bonding heterodesmic interactions predicted by CCDC. The overall conclusion drawn is that the ability to attain solubility is the result of lower intermolecular hydrogen-bond network crystallinity and a degree of amorphization.

### 3.6. Thermal Behavior and Stability Analysis of Rebamipide and Selected DEM Using DSC and TGA

**Figure 8:**
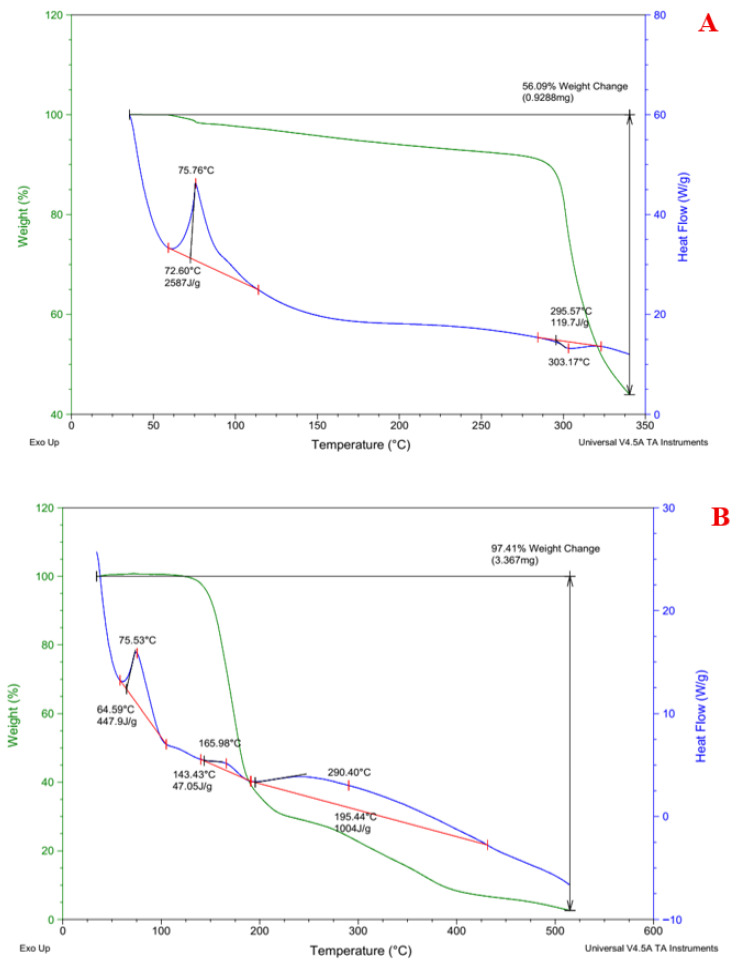
DSC (blue line) and TGA (green line) of (**A**) pure Rebamipide compared to (**B**) the selected DEM (1:3:1).

To evaluate possible alterations in structure and improvements in stability, thermal property analysis using DSC and TGA was performed on pure Rebamipide and the chosen DEM formulation (1:3:1 Rebamipide:Malonic acid:Urea) as shown in Figure 8. As reported in [37], Pure Rebamipide had a sharp endothermic melting peak at ~303.17 °C (ΔH = 119.7 J/g), which reflects the crystal-like solid to liquid transition point. It also had a smaller dehydration event around ~75.76 °C (ΔH = 25.87 J/g). On the contrary, a thermogram bearing multiple broad endothermic transitions was revealed, the first around ~75.53 °C (ΔH = 447.9 J/g), the next at ~165.98 °C (ΔH = 47.05 J/g), and a high-temperature transition at ~290.40 °C (ΔH = 1004 J/g). With the DEM’s disappearance of Rebamipide’s sharp melting peak and the presence of broad transitions, disruption of the crystalline lattice is indicated [38]. This suggests that strong intermolecular interactions with the eutectic components may be responsible for the partial or complete amorphization.

Meanwhile, thermogravimetric analysis showed that under similar conditions, pure Rebamipide lost weight in a single step, reaching a total loss of 56.09% post 250 °C, while at 165 °C, the DEM showed significant weight loss in a multi-step degradation pattern and 97.41% overall weight change, which extended beyond 290 °C. Sequentially decomposing, hydrogen-bonded complexes in a multicomponent system caused the broadened decomposition profile of the DEM, which also indicates loss of thermal stability when compared to the uncontaminated medications [39]. All aspects of CCDC’s predictions correlate with DSC and TGA. The absence of distinct melting peaks, broad shifts to thermal events, and multi-step degradation all suggest extensive hydrogen bonding and disruption of the crystalline lattice. The 1:3:1 DEM showed a significant increase in solubility due to higher levels of amorphization and increased molecular mobility in the compound, as also confirmed via spectroscopy.

### 3.7. Powder X-Ray Diffraction (PXRD) Analysis of Rebamipide and Selected DEM

**Figure 9:**
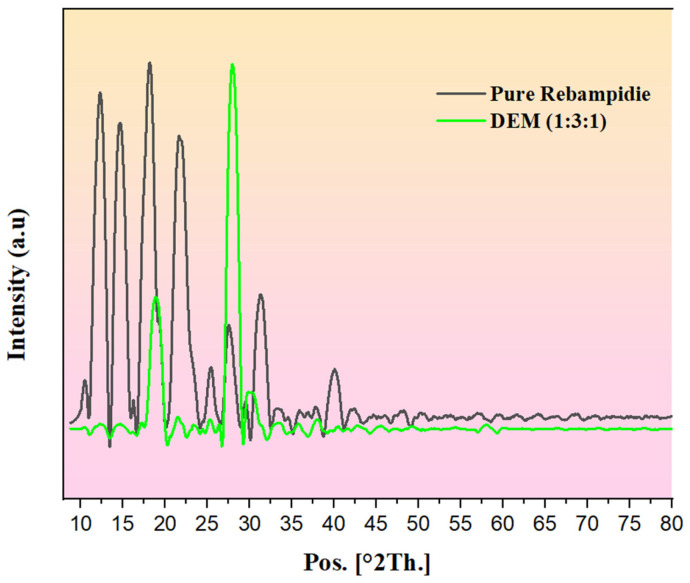
PXRD pattern of pure Rebamipide compared to the selected DEM (1:3:1).

The pure Rebamipide PXRD diffractogram possesses several sharp, intense peaks at 20 = 10–30 and proves that the compound is highly crystalline [40]. A decrease in the intensity of peaks, a partial loss of the characteristic crystalline facets of Rebamipide, and the formation of broad diffuse halos were reported in the selected ratio of 1:3:1, that is, (Rebamipide:Malonic Acid:Urea DEM), respectively (Figure 9). This confirms a reduction in crystalline order and partial amorphization of Rebamipide within the DEM matrix [41]. The reduced crystallinity observed in the PXRD results correlates with the predicted hydrogen bonding network from the CCDC, which notes the presence of multiple short linear interactions, suggesting hydrogen bonding with Rebamipide and both eutectic components.

It is obvious that these strong intermolecular hydrogen bonds are responsible for disrupting the Rebamipide crystal lattice, as evident by the FTIR spectral shifts, particularly the broadening of the 3400–3200 cm^−1^ region, as well as the C=O band downshift to 1652 cm^−1^. The structural disruption observed in the saturated solubility studies is directly linked to the enhanced aqueous solubility of the 1:3:1 DEM. The extent of molecular dissolution rate and mobility is associated with the hydrogen bonding dominance in the amorphous-like state. The PXRD, FTIR, and the predictive models in conjunction are definitive in showing the eutectic formulation technique employed and the resultant hydrogen bond-driven lattice destabilization resulted in a transformation of Rebamipide to a less crystalline and therefore, more soluble state.

### 3.8. Selection of the Optimal DEM Formulation

Every analytical method suggests that the 1:3:1 DEM is the best formulation. A definitive mechanism is established with the combination of predictions made on the crystallographic database (CCDC), spectral analysis (FTIR), crystallinity evaluations (PXRD), and thermal stability assessments (DSC, TGA). The disruptive crystallinity lattice amorphization and hydrogen bonding networks enhance the solubility of Rebamipide. Such transformations in the structure explain why the selected DEM performs best and is thus the best option for further formulation as bioadhesive controlled-release granules.

The mechanistic role of the optimized deep eutectic mixture is best understood through the integration of computational, solid-state, and functional evidence. At the molecular level, CSD-based in silico analysis predicted multiple favorable hydrogen-bonding interactions between Rebamipide, malonic acid, and urea, characterized by high interaction frequencies and favorable geometric parameters. These predictions were experimentally substantiated by FTIR spectral shifts, DSC thermal transitions, PXRD peak attenuation, and altered TGA profiles, collectively indicating disruption of the crystalline lattice and partial amorphization of Rebamipide within the eutectic system. The superior performance of the selected DEM also retrospectively validates the component-selection strategy, confirming that the combined use of urea and malonic acid was more effective in promoting solubility enhancement and solid-state modification than would be expected from less cooperative eutectic-forming alternatives. Subsequent in vitro, ex vivo, and in vivo findings further demonstrate that this molecular reorganization translates into improved functional performance, including sustained dissolution, enhanced mucoadhesion, prolonged gastric retention, and controlled drug release.

### 3.9. Preparation of Rebamipide-Loaded DEM as Bioadhesive Controlled-Release Granules

The fabrication of bioadhesive controlled-release granules was done using a DEM (Rebamipide:Malonic acid:Urea) in the ratio of 1:3:1, following computational modeling (CCDC) and experimental aqueous solubility data. In the wet granulation, the hypothesis was confirmed by the obtained free-flowing granule system. This DEM kept its homogeneous state throughout the granulation, signifying that the processing did not break the eutectic structure. FTIR studies suggest that the granules will likely preserve the hydrogen bonding interactions of Rebamipide, urea, and malonic acid, vital in retaining the enhanced solubility recorded in the preformulation stage. In addition, the raw DEM PXRD outcome showed an amorphous structure, which is ideal in controlled-release formulations. Amorphous structures permit a greater rate of dissolution and thus, a greater potential range in release kinetics that can be controlled.

Viewed from a technology standpoint, the granules exhibited satisfactory compressibility and low friability, which is required for capsule filling. Incorporating bioadhesive polymers into the formulation is expected to increase time spent in the stomach, allowing for the localized delivery of the drug and potentially improving the drug’s oral bioavailability through prolonged gastric residence and enhanced dissolution, although formal pharmacokinetic studies are required to confirm this [42]. The solubility-enhancing DEM and controlled-release matrix are likely to offer two benefits: the drug will dissolve quickly at the initial stage since it is in an amorphous state, and the bioadhesive system will allow the drug to release gradually.

### 3.10. Physical Characterization of Prepared Granules

**Figure 10:**
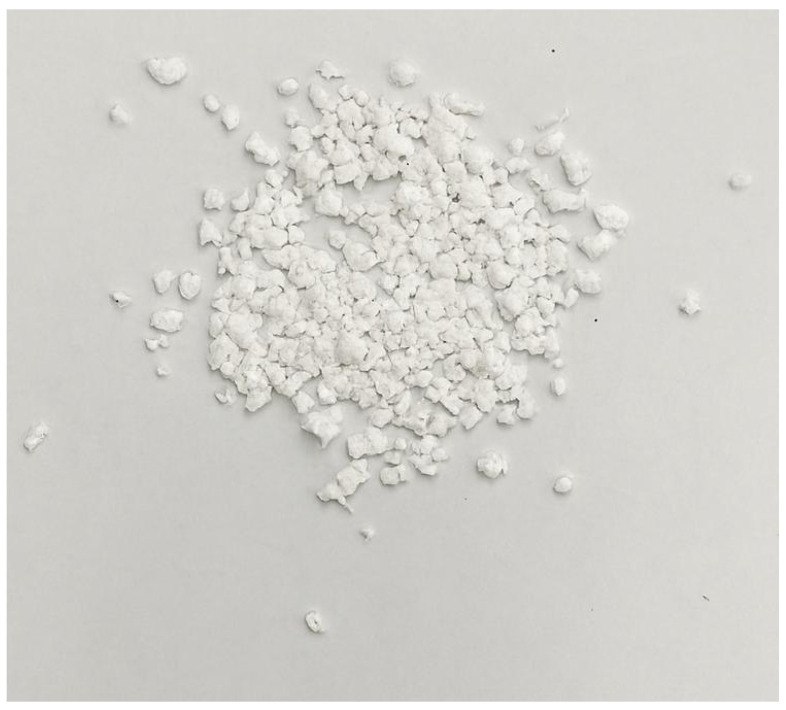
The uniform white appearance of the prepared Rebamipide-loaded deep eutectic mixture (DEM) granules.

The average mixing proportion of the components of the deep eutectic mixture to be employed in carrying out the controlled experimentation was 1:3:1 (Rebamipide:Malonic acid:Urea), as shown in Figure 10. The experiment was conducted, and the white homogeneous granules of the optimal size were observed, which confirms that all the stages in wet granule formation up to drying had been carried out successfully. The relatively moist granules (in the experiment), as viewed from a pharmaceutical perspective, mean that the product will not be influenced by either of the dry or storage conditions.

The granules provided minimal friability, confirming the sufficient strength and resistance of the material to breakage during transport and processing. The angle of repose gave a good measure of the flow characteristics and indicated that the granules demonstrated a critical free-flowing behavior, which is necessary for uniform weights and accurate dosing in capsule preparation [43]. The existence of urea and malonic acid in the DEM matrix likely explains these results by aiding in the formation of a smooth surface morphology and reducing interparticle interaction [44]. Overall, the physical evaluation (Table 5) showed that the DEM granules adequately and seamlessly incorporated the desired mechanical integrity, flowability, and stability appropriate for encapsulation, demonstrating the intended formulation for the bioadhesive, controlled-release capsules. The combined physical characteristics and the increased solubility of the DEM (saturated solubility, FTIR, and CCDC data) indicate that a good starting point exists for the upcoming in vitro release and bioadhesion tests.

### 3.11. Swelling Index and Matrix Erosion

**Figure 11:**
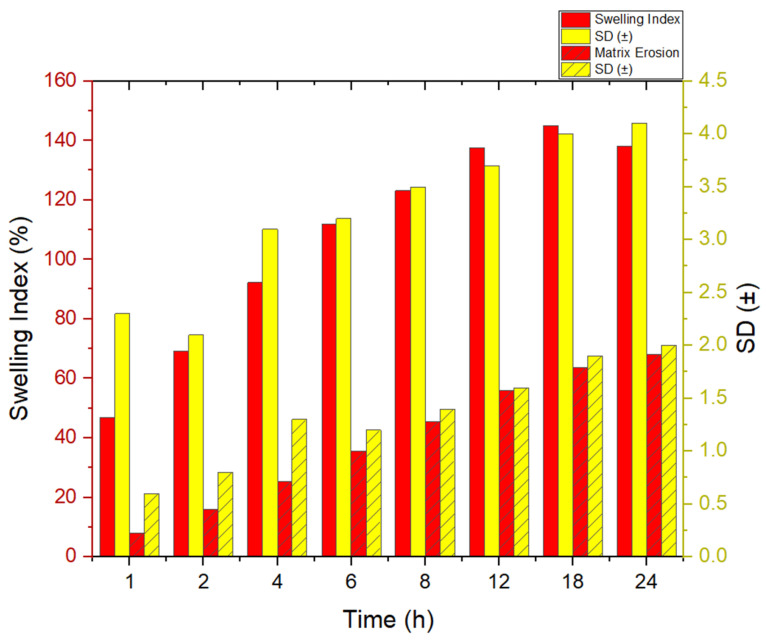
The swelling index and matrix erosion of Rebamipide-loaded DEM granules over 24 h.

The swelling index and matrix erosion of Rebamipide loaded with DEM granules were conducted over 24 h in simulated gastric fluid (0.1 N HCl, pH 1.2) at 37 ± 0.5 °C (See Figure 11). The initial few hours saw a rapid increase in granule swelling, ranging between 46 percent after one hour and up to 92 percent after four hours. Swelling and hydration of the granules occurred to a maximum of about 144 percent at eighteen hours, and after 24 hours, it decreased to about 140 percent. This is likely a systematic softening and erosion of the matrix, or a partial failure as a result of overhydration, which causes relaxation of the structure [45]. The observation implies the existence of larger structural units of the matrix made of reactive granules and a highly porous and hydrophilic polymer. By the end of work, a substantial change in the granule matrix erosion (~8–~69%) was noted (between one hour and twenty-four hours). The first 4 h showed very slow erosion (≤25% of 4 h), which suggests that the initial drug release may occur primarily through diffusion. After 8 h, polymer erosion was rapid, which suggests that a significant part of the drug is released through polymer disintegration, and erosion is clearly associated with diffusion [46]. The swelling curve progressed before the erosion curve, and maximum swelling occurred before peak erosion. This shows that granule hydration and gel layer formation occurred early, providing structural integrity and mucoadhesive potential, while erosion dominated in the later stages, allowing complete release of the drug [47]. The sustained swelling and balanced controlled erosion provide prolonged gastric retention and controlled-release properties [48]. These results confirm that the granule formulation sufficiently holds its structure for sustained delivery, while the gradual erosion of the formulation provides complete delivery in the expected time frame. Such a swelling–erosion relationship is mechanistically critical for once-daily mucoadhesive gastroretentive formulations, as it ensures both prolonged gastric retention and sustained drug release through a coordinated diffusion–erosion mechanism. The temporal relationship between swelling and matrix erosion provides important mechanistic insight into the drug release behavior of the DEM granules. The initial rapid swelling phase reflects polymer hydration and gel layer formation, which facilitates the diffusion of Rebamipide through the hydrated matrix. As the system progresses, matrix erosion becomes increasingly significant, contributing to the release of drug entrapped within the inner matrix structure. This sequential transition from swelling-dominated diffusion to erosion-controlled release supports a dual drug release mechanism. This interpretation is further corroborated by kinetic modeling, where the Korsmeyer–Peppas exponent (*n* ≈ 0.47) indicates anomalous (non-Fickian) transport involving both diffusion and polymer relaxation/erosion. From a formulation perspective, this behavior is highly advantageous, as early swelling enhances mucoadhesion and gastric retention, while controlled matrix erosion ensures sustained drug release over 24 h, supporting the development of once-daily gastroretentive delivery systems.

### 3.12. In Vitro Dissolution Study

**Figure 12:**
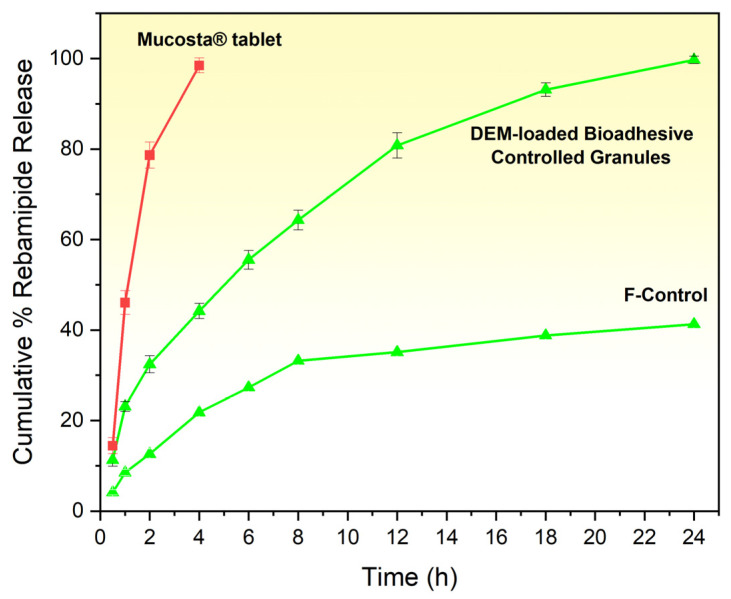
In vitro release profile of Rebamipide-loaded DEM granules, DEM-free matrix control granules (F-Control), and the marketed tablet (Mucosta^®^ 100 mg) over 24 h in 0.1 N HCl pH 1.2 (mean ± SD, *n* = 3).

All quantitative measurements were performed using a validated analytical method, ensuring the reliability and reproducibility of the obtained data. The in vitro dissolution profiles of Rebamipide from DEM granule-loaded capsules, F-Control granules, and the marketed tablet were compared in 0.1 N HCl (pH 1.2) at 37 ± 0.5 °C (Figure 12). The prepared DEM granules exhibited a gradual and sustained drug release over 24 h, with an initial release of 11.3 ± 1.4% at 0.5 h, reaching 44.2 ± 1.7% at 4 h, and achieving near-complete release (99.7 ± 0.8%) by 24 h. In contrast, the marketed tablet showed a rapid drug release, with 35.5 ± 1.3% released at 0.5 h, 82.2 ± 2.1% at 2 h, and almost complete release (96.6 ± 1.2%) by 4 h, after which no further significant release was observed.

F-Control granules exhibited substantially lower and incomplete dissolution: 4.1 ± 0.6% at 0.5 h, 7.3 ± 0.8% at 1 h, 12.6 ± 1.1% at 2 h, 21.8 ± 1.4% at 4 h, 28.4 ± 1.8% at 6 h, 33.2 ± 2.1% at 8 h, 37.9 ± 1.7% at 12 h, and reaching a plateau of 41.3 ± 2.3% at 24 h, confirming that the polymer matrix alone cannot overcome the solubility limitation of crystalline Rebamipide.

The sustained-release behavior of the optimized DEM granules arises from the combined contributions of the eutectic microenvironment and the polymeric release matrix. At the molecular level, the Rebamipide–malonic acid–urea eutectic system forms a cooperative hydrogen-bonding network that reduces drug crystallinity, enhances molecular dispersion, and increases the apparent thermodynamic activity of Rebamipide, thereby overcoming the intrinsic dissolution limitation of the crystalline drug. At the formulation level, HPMC K100M forms a hydrated gel barrier upon contact with gastric medium, while chitosan contributes both matrix integrity and mucoadhesive character. Together, these polymers delay medium penetration and drug escape, thereby modulating release from an initially diffusion-dominant phase to a later erosion-assisted phase. Thus, the DEM primarily governs solubility enhancement, whereas the polymeric matrix governs temporal release control, and the interplay of both components accounts for the observed near-complete yet prolonged dissolution profile over 24 h.

The dissolution profiles clearly demonstrate that the optimized DEM granules behave as a solubility-enhanced controlled-release system, whereas the marketed tablet exhibits conventional immediate-release behavior. This distinction arises because the DEM granules combine hydrogen-bond-mediated amorphization and solubility enhancement with polymer-regulated release retardation, while the marketed tablet lacks both the eutectic solubilization microenvironment and the diffusion–erosion matrix required for sustained release. Difficulties in the initial phases of release from the DEM granules can stem from the constituent predominant network of hydrogen bonding between Rebamipide, malonic acid, and urea in the eutectic matrix. This is significant from the CCDC and solid-state characterization studies of FTIR, DSC, TGA, and PXRD, and is presumably helpful for reducing the immediate wettability and the dissolution rate of the drug [49]. Furthermore, the presence of HPMC K100M and chitosan in the granule matrix also incorporated an extra gel-forming and bioadhesive barrier that also influenced drug release [50,51]. The marketed, non-granule tablet, which lacks the sustained release and bioadhesive components, had a complete ‘dissolution’ of the drug in less than 4 h. The DEM granules, however, could ‘dissolve’ the drug throughout for the substantially longer, more clinically relevant period of up to 24 h. This sustained release means that the granule is far more clinically relevant than the marketed tablet, which helps minimize therapeutic drug levels and enables maintaining therapeutic levels of the drug up to 24 h

Kinetic evaluation showed that the DEM granules reached a T_50_% of 5.03 h, while the marketed tablet reached 0.82 h, confirming the extended-release ability of the eutectic-based system. Over the study period, the granule dissolution efficiency, DE%, was 71.78% compared to the tablet’s 68.16%. The similarity factor, f_2_, of 18.82 demonstrated that the release profile was substantially different. To quantitatively elucidate the release mechanism, the dissolution data were fitted to multiple kinetic models, including zero-order, first-order, Higuchi, and Korsmeyer–Peppas models. The Higuchi model demonstrated the highest correlation coefficient (R^2^ = 0.9966), indicating that drug release is predominantly governed by diffusion through a hydrated polymeric matrix. Furthermore, the Korsmeyer–Peppas model yielded a release exponent (*n* ≈ 0.47), which falls within the range of 0.43 < *n* < 0.85 for spherical systems, confirming anomalous (non-Fickian) transport. This indicates a coupled mechanism involving both diffusion and polymer relaxation/erosion. These findings are consistent with the swelling–erosion behavior of the HPMC K100M/chitosan system and demonstrate that the sustained-release profile of the DEM granules is quantitatively governed by a diffusion–erosion mechanism. In contrast, the marketed formulation displayed rapid immediate-release behavior, reaching near-complete release within approximately 4 h, without evidence of matrix-mediated release control [52]. The enhanced mucoadhesive behavior of the DEM granules further complements this release mechanism by prolonging gastric residence time, thereby maintaining continuous exposure of the hydrated matrix to the dissolution medium. This sustained interfacial contact supports diffusion-controlled drug release, consistent with the Higuchi model and anomalous transport behavior observed in kinetic analysis. The observed dissolution behavior aligns with the enhanced aqueous solubility of the 1:3:1 DEM from the saturated solubility studies and the strong intermolecular hydrogen bonding predicted by CCDC simulations and confirmed by FTIR, DSC, TGA, and PXRD. This combination of molecular-level interaction and polymeric release control explains both the improved solubility and the sustained release profile.

To verify the indispensable role of the DEM in achieving the observed dissolution performance, a DEM-free matrix control formulation (F-Control)—containing pure crystalline Rebamipide in an identical HPMC K100M/chitosan/MCC matrix—was evaluated under the same conditions. F-Control granules reached a dissolution plateau of 41.3 ± 2.3% cumulative release by 24 h, in stark contrast to the near-complete release of DEM granules (99.7 ± 0.8%) over the same period (Figure 12). This plateau behavior is mechanistically consistent with the solubility-limited dissolution of crystalline Rebamipide (intrinsic aqueous solubility: 0.045 mg/mL): despite HPMC K100M gel formation providing a sustained-release barrier and chitosan-mediated mucoadhesion maintaining gastric contact, the low thermodynamic activity of crystalline Rebamipide restricts the concentration gradient driving diffusion, resulting in incomplete release regardless of contact time. In contrast, the DEM’s amorphization of Rebamipide—confirmed by PXRD and DSC—and its cooperative hydrogen-bond solubilization network elevate the effective drug activity in the matrix microenvironment, providing the thermodynamic driving force for near-complete 24 h release. Kinetic analysis further distinguishes the two formulations: F-Control exhibited dissolution efficiency DE% = 28.7%, T_50_% not reached within 24 h, and a Korsmeyer–Peppas *n* = 0.38 consistent with Fickian diffusion from a solubility-limited matrix; DEM granules achieved DE% = 71.78%, T_50_% = 5.03 h, and *n* = 0.47 (anomalous transport), confirming that the DEM fundamentally transforms the release mechanism from solubility-limited diffusion into a complete, polymer-modulated controlled-release system. These results unambiguously establish that the HPMC/chitosan matrix alone cannot replicate the dissolution performance of DEM granules, validating the eutectic system as the essential driver of both solubility enhancement and near-complete controlled release.

The dissolution data for F-Control confirmed the solubility-limited behavior of crystalline Rebamipide within the HPMC K100M/chitosan matrix. F-Control granules reached a plateau of 41.3 ± 2.3% cumulative release by 24 h (DE% = 28.7%, Korsmeyer–Peppas *n* = 0.38), consistent with Fickian diffusion from a matrix in which drug release is constrained by the low intrinsic aqueous solubility of crystalline Rebamipide (0.045 mg/mL). Despite sustained gel-layer formation by HPMC K100M and mucoadhesive contact maintained by chitosan, the low thermodynamic activity of the crystalline drug restricted the concentration gradient driving diffusion, resulting in incomplete release regardless of contact time. These findings confirm that the polymer matrix alone is insufficient to overcome the solubility barrier of Rebamipide, and establish the indispensable role of the DEM in achieving the near-complete (99.7 ± 0.8%) controlled release observed in the optimized formulation

The enhanced dissolution and prolonged release behavior of the DEM granules are expected to improve the oral bioavailability of Rebamipide by increasing drug availability in the gastric environment and maintaining sustained exposure at the absorption site. These effects are particularly relevant for poorly soluble drugs, where dissolution is the rate-limiting step for absorption. However, it should be noted that bioavailability enhancement was not directly evaluated in the present study.

### 3.13. Ex Vivo Bioadhesive Strength Determination

**Figure 13:**
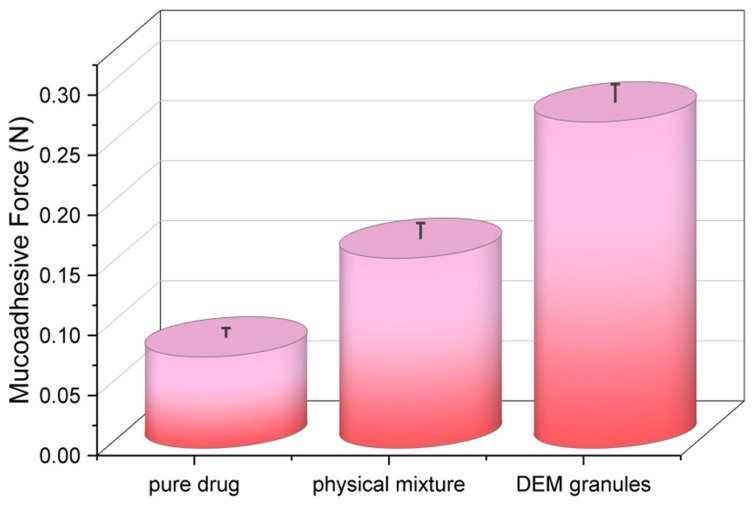
Ex vivo bioadhesive strength of Rebamipide-loaded DEM granules compared to pure drug and physical mixture.

The bioadhesive strength of the pure drug, its physical mixture, and the optimized deep eutectic mixture (DEM) granules were quantitatively evaluated using freshly excised sheep stomach mucosa as the biological substrate. The results (Figure 13) showed a clear and statistically significant (*p* < 0.05) enhancement in bioadhesive force for DEM granules (0.29 ± 0.02 N) compared to both the physical mixture (0.19 ± 0.01 N) and the pure drug (0.10 ± 0.01 N). A ~2.9-fold increase from the solitary medication indicates the combined effect of the DEM system and the chitosan (cationic mucoadhesive polymer) role in gastric mucosa adhesion [53]. Increased adhesion could be enhanced by the granules’ hydration and swelling, which aid in polymer chain interpenetrating the mucin layer. Furthermore, the ionic chitosan polymer bonds and electrostatic attractions between the chitosan amino groups and gastric mucus’s negatively charged sialic acid subsequences [54]. Mechanistically, the granule system mucoadhesion explains the docking predictions and the FTIR/DSC data, which marked strong hydrogen bond formation within the DEM system and excipients.

The enhanced mucoadhesive strength of the DEM granules can be mechanistically attributed to the synergistic interplay between chitosan-mediated electrostatic interactions and the hydrogen-bonding network introduced by the deep eutectic system. The protonated amino groups of chitosan establish strong electrostatic interactions with negatively charged sialic acid residues present in gastric mucin, while the DEM components contribute additional hydrogen-bonding interactions that improve surface wettability and hydration of the granules. This dual interaction mechanism facilitates polymer chain interpenetration into the mucus layer and strengthens interfacial adhesion. The resulting increase in bioadhesive force (0.29 ± 0.02 N) reflects this cooperative effect. Importantly, enhanced mucoadhesion directly contributes to prolonged gastric residence, as confirmed by radiographic observations, which in turn supports sustained drug release by maintaining continuous contact between the formulation and the gastric environment.

As such, a prolonged gastric residence time is expected to improve sustained drug release, as the in vitro dissolution studies indicate. The choice of sheep stomach mucosa also added to the findings’ physiological relevance because it matches the human gastric mucosa in both composition and texture [55]. The sustained release profile for which 99.7% of Rebamipide was released over 24 h in vitro is indicative of high bioadhesive strength. The strong adhesion prolonged gastrointestinal residence time, which increases local drug concentration while maintaining a diffusion-driven release mechanism, as confirmed by kinetic analysis.

### 3.14. In Vivo Radiographic Evaluation

**Figure 14:**
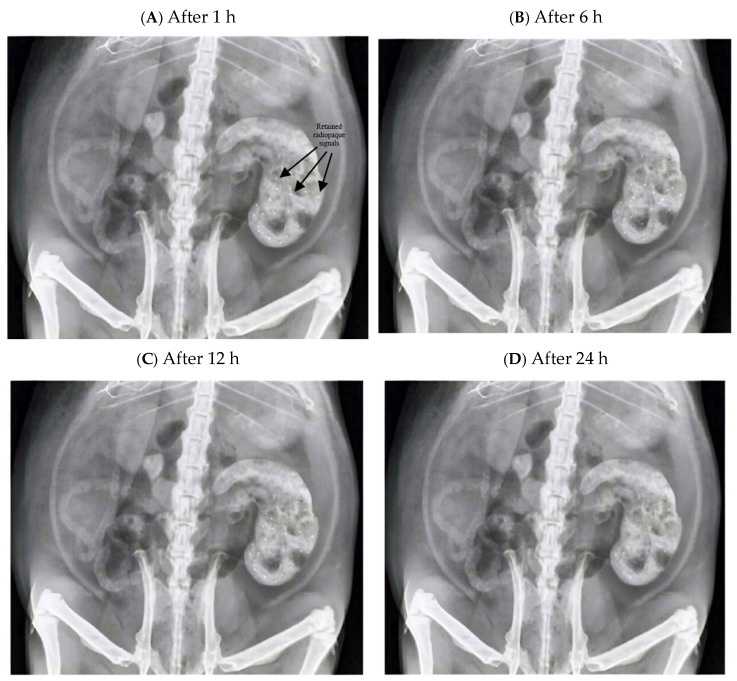
Abdominal X-ray images of male Sprague–Dawley rats after oral administration of BaSO_4_-loaded Rebamipide–DEM granules at 1, 6, 12, and 24 h, demonstrating sustained gastric retention, (The gastric region indicated with arrows after 1 h to facilitate identification of radiopaque signals corresponding to the administered formulation).

Radiographic observations of BaSO_4_-loaded Rebamipide–DEM granules showed that these granules featured maintained gastric retention in male Sprague–Dawley rats within the 24 h observation period (Figure 14). One hour post administration, granules within the stomach showed successful dispersion as evidenced by the multiple radiopaque patches within the gastric cavity (Figure 14A). The granules showed good localization, which suggests that they rapidly hydrated and swelled sufficiently to avoid intestinal transfer. At 6 h (Figure 14B), most of the granules remained visible, opaque to radiation, and present in the stomach, with the expected reduction in the concentration of radiopaque granules that suggests matrix erosion of the granules. This retention pattern and the still permissible release proved the bioadhesive capacity of the formulation to the stomach. After 12 h (Figure 14C), there remained passively radiographed granules, albeit with a much lower concentration and visible outline. This suggests sustained matrix erosion and release of Rebamipide, explaining the loss of radiographic signal. In Figure 14, the gastric anatomical landmarks visible on the radiographs (gastric fundus, body, and pyloric region) were used to confirm granule localization within the stomach at each time point. The progressive decline in radiopaque signal intensity from 1 h to 24 h is consistent with concurrent matrix erosion and drug release rather than gastric emptying, which is further supported by the in vitro swelling–erosion data. It should be noted that a direct in vivo radiographic comparison with the commercially available Rebamipide tablet was not performed in this study due to study design constraints; the marketed tablet (Mucosta^®^, 100 mg) lacks a radiopaque component and would require BaSO_4_ supplementation for radiographic visualization. This represents a limitation of the current work, and future studies are recommended to include such a comparative arm to fully substantiate the gastroretentive advantage of the DEM granule system. Lower concentration and visible outline. This suggests sustained matrix erosion and release of Rebamipide, explaining the loss of radiographic signal.

The progressive decline in radiopaque signal density from 1 h to 24 h provides radiographic evidence of sustained gastric retention, consistent with the prolonged bioadhesive properties measured ex vivo. At 24 h, Figure 14D, within the gastric region, faint but clear radiopaque signals were positioned where the DEM granules were radiographically visible, again confirming the retention of the DEM granules within the gastric region. This clearance retention suggests the potential to extend dosing to once a day. The radiographic findings corresponded closely with the in vitro dissolution and swelling/erosion profiles. The sustained release behavior observed in vitro, where >90% release occurred by 24 h, explains the sustained release behavior in radiographs. The decrease in radiopacity sustained behavior is explained by the swelling and matrix erosion mechanism. The 24 h gastric retention corresponds with the improved bioadhesion demonstrated in the ex vivo sheep stomach models, where DEM granules were significantly stronger mucoadhesive than the pure Rebamipide and physical mixtures that incorporated DEM, which arise from the combined effects of chitosan-mediated electrostatic adhesion and DEM-induced hydrogen bonding, thereby supporting sustained drug release kinetics. These combined findings indicate that the Rebamipide–DEM bioadhesive controlled-release granule system achieves prolonged gastric residence, which is expected to support localized mucosal drug delivery and improve therapeutic outcomes in ulcer management. Formal in vivo pharmacokinetic studies are warranted to quantitatively confirm the anticipated bioavailability improvement.

Despite the encouraging in vivo radiographic evidence of prolonged gastric residence, these findings should be interpreted as proof of gastroretentive behavior rather than direct evidence of clinical efficacy. The present study did not evaluate systemic pharmacokinetics, gastric mucosal pharmacodynamics, dose–exposure relationships, long-term formulation stability, or human in vivo performance. Therefore, while the radiographic data substantiate formulation retention under preclinical conditions, further pharmacokinetic and efficacy-oriented studies are required to determine whether the observed gastroretentive and controlled-release properties translate into meaningful therapeutic benefit.

Earlier attempts at formulations that try to overcome the low solubility and rapid clearance of Rebamipide essentially revolved around solid dispersions, cocrystals, and nanoparticle-based formulations. Solid dispersions raised the dissolution rate but were limited by physical instability as well as drug recrystallization during storage. Cocrystal techniques increased solubility moderately but suffered from issues of scalability and reproducibility, performing inconsistently depending on the co-former. The products were nanoparticle and liposomal and showed heightened systemic absorption but limited control of gastric residence, and had complex production processes. Floating gastroretentive tablets and gels produced increased gastric retention, but these products commonly had burst release and unsatisfactory solubility enhancement. In comparison, the current DEM-based bioadhesive controlled-release granules presented a twofold advantage: a 220-fold solubilization enhancement (10.08 mg/mL vs. 0.045 mg/mL for plain drug) with prolonged gastric retention for a long duration (up to 24 h). Combinatorial amorphization through hydrogen bonding and mucoadhesive polymer control led to extended release (T_50_% ~5 h compared with 0.8 h for commercial tablets), optimal bioadhesion (~3 times greater than plain drug), and once-daily dose capability. Consequently, the DEM-based innovation is deemed highly scalable, robust, and quite viable compared to the other approaches discussed earlier. The observed prolonged gastric residence of the formulation may further contribute to enhanced drug absorption by extending the residence time at the absorption site. Nevertheless, direct pharmacokinetic studies are required to confirm this effect.

## 4. Conclusions

This study demonstrates, through an integrated mechanistic framework, the successful design and evaluation of Rebamipide-loaded deep eutectic mixture (DEM) granules for gastroretentive controlled delivery. In silico CCDC analysis first predicted favorable hydrogen-bonding interactions between Rebamipide, malonic acid, and urea, which were subsequently supported by FTIR, DSC, TGA, and PXRD findings indicating reduced crystallinity and partial amorphization of the drug. The analytical method used for quantification was validated, supporting the reliability of solubility and dissolution data presented in this study. These solid-state modifications mechanistically explain the marked enhancement in solubility observed for the optimized 1:3:1 DEM system. Functional validation at multiple levels further showed that the optimized granules achieved sustained drug release, significant mucoadhesive strength, and prolonged gastric retention, thereby establishing a coherent link between molecular interaction, physicochemical transformation, and formulation performance.

The in vitro design showed drug release over a 24 h period, along with the swelling and erosion of the granules, erosion control, and proposed strength of mucoadhesion, which surpassed that of the pure drug and other physical combinations. Quantitative kinetic analysis confirmed that this prolonged release profile was governed by anomalous diffusion–erosion transport within the HPMC K100M/chitosan matrix, whereas the marketed product retained the rapid immediate-release characteristics of a conventional tablet. Dissolution comparison with DEM-free matrix control granules (F-Control) confirmed that the polymer matrix alone achieved only ~41% release by 24 h due to the solubility limitation of crystalline Rebamipide, establishing the indispensable role of the eutectic system in achieving near-complete (99.7%) controlled release and validating the DEM as the essential driver of both solubility enhancement and release performance. These findings collectively suggest that the formulation is expected to improve drug retention time in the stomach and increase local drug availability for gastroretentive treatment. Nevertheless, the current findings remain preclinical in nature, and translation into clinical efficacy is limited by the absence of pharmacokinetic, pharmacodynamic, long-term stability, scale-up, and human validation data.

Gastric retention of BaSO_4_-loaded DEM granules was demonstrated radiographically in rats over a 24 h observation period, with progressive reduction in radiopaque signal consistent with sustained matrix erosion and controlled drug release. This is in contrast to the expected rapid gastric emptying associated with conventional immediate-release tablets. The persistence of granules within the gastric cavity highlights the synergistic contributions of the DEM system with bioadhesive polymers to achieve gastric retention and controlled release. Thus, Rebamipide-loaded DEM granules describe a promising gastroretentive delivery platform that addresses the challenges of prolonged gastric residence, local sustained release, and poor aqueous solubility. The combination of in silico, in vitro, ex vivo, and in vivo approaches taken provides a strong rationale for advancing this formulation into preclinical pharmacokinetic and clinical studies to improve the treatment of gastric ulcers and other gastrointestinal disorders.

## Figures and Tables

**Table 1:** Rebamipide, urea, and malonic acid functional groups as queries in the ConQuest software for predictions of hydrogen bonding probabilities at the intermolecular level.

Rebamipide	Urea	Malonic Acid
C–Cl (aryl chloride)	NH_2_	Hydroxyl
O25	NH_2_	Hydroxy
O4	NH_2_	Hydroxy
O16	NH_2_	Hydroxy
NH17	carbonyl	carbonyl
OH5	carbonyl	carbonyl
NH6	carbonyl	carbonyl

**Table 2:** The composition of capsule-loaded DEM granules of Rebamipide.

Component	Function	Quantity (mg)
Selected DEM (contains 100 mg Rebamipide)	Solubilized drug matrix	200
Chitosan (high-viscosity grade)	Bioadhesive polymer	45
HPMC K100M	Sustained release matrix	90
Microcrystalline Cellulose (MCC)	Filler and flow enhancer	134
Aerosil	Anti-caking agent	7
Magnesium Stearate	Lubricant	14
Talc	Glidant	9.5
Total (capsule size 0)		500

**Table 3:** The hydrogen bond angles and distances pertaining to the functional groups of malonic acid and Rebamipide, as well as the bonded predicted intermolecular hydrogen bonds in the CSD model, are documented in Table 3 (model means ± SD).

Donor–Acceptor Pair	Hits	H-Bond Length (Å)	H-Bond Angle (°)
OH5—C=O of Malonic acid	7033	1.885 ± 0.345	163.34 ± 21.51
NH17—C=O of Malonic acid	351	2.212 ± 0.239	155.81 ± 16.75
O25—OH of Malonic acid	394	1.808 ± 0.253	161.85 ± 18.24
C–Cl (aryl chloride)—OH	21	2.872 ± 0.121	105.87 ± 21.36

**Table 4:** With respect to the CSD hydrogen bond predicted hits (models ± SD), we have included the cor-responding angles and distances for the urea and Ta returning functional groups and Rebamipide, with the intermolecular hydrogen bond hits predicted in the CSD model (model means ± SD).

Donor–Acceptor Pair	Hits	H-Bond Length (Å)	H-Bond Angle (°)
O25—NH_2_ of Urea	4311	2.131 ± 0.204	158.626 ± 14.389
OH5—C=O of Urea	333	1.768 ± 0.326	162.73 ± 15.88
NH17—C=O of Urea	841	2.131 ± 0.198	156.56 ± 14.19
O16—NH_2_ of Urea	50	2.025 ± 0.129	166.60 ± 10.73
O4—NH_2_ of Urea	1870	2.192 ± 0.259	155.32 ± 19.69

**Table 5:** Physical characterization of Rebamipide-loaded DEM bioadhesive controlled-release granules (mean ± SD, *n* = 3).

Parameter	Result (Mean ± SD, *n* = 3)	Acceptance Criteria
Angle of repose (°)	28.4 ± 1.2	<30° (excellent flow)
Bulk density (g/mL)	0.412 ± 0.018	Reported value
Tapped density (g/mL)	0.487 ± 0.021	Reported value
Carr’s compressibility index (%)	15.4 ± 0.9	≤15–25% (good–fair flow)
Hausner’s ratio	1.18 ± 0.02	1.00–1.25 (good flow)
Loss on drying/moisture content (%)	1.87 ± 0.14	≤2.0%
Friability (%)	0.43 ± 0.07	<1.0% (USP <1216>)
Mean particle size (µm)	412.6 ± 28.3	300–600 µm (capsule fill)
Particle size distribution (span)	0.74 ± 0.06	<1.0 (narrow distribution)
Drug content uniformity (%)	98.7 ± 1.2	85–115% (USP <905>)

## Data Availability

The original contributions presented in this study are included in the article. Further inquiries can be directed to the corresponding author.
